# Knockdown of Gas6 Exerts Anti-Esophageal Cancer Effects by Inhibiting the PI3K/AKT Pathway

**DOI:** 10.3390/cimb46100676

**Published:** 2024-10-13

**Authors:** Shuang Gao, Yu Wang, Ming Huang, Jianxin Guo, Zhongbing Wu, Jing Li

**Affiliations:** 1 College of Integrated Chinese and Western Medicine, Hebei Medical University, Shijiazhuang 050017, China; 20211082@stu.hebmu.edu.cn (S.G.); 23031100064@stu.hebmu.edu.cn (Y.W.); 22031100077@stu.hebmu.edu.cn (M.H.); 22033100254@stu.hebmu.edu.cn (J.G.); 2 Institute of Integrated Traditional Chinese and Western Medicine, Hebei Medical University, Shijiazhuang 050017, China

**Keywords:** ESCC, Gas6, PI3K, migration, invasion

## Abstract

Esophageal squamous cell carcinoma (ESCC) is a malignant tumor of the digestive tract with strong migratory and invasive abilities. Gas6 is closely associated with the progression of many malignant tumors; however, the role of Gas6 in the progression of esophageal cancer is unclear. Here, we report that the knockdown of Gas6 inhibited esophageal cancer cell proliferation, migration, and invasion. In addition, Gas6 knockdown downregulated the levels of P-PI3K and P-AKT. Taken together, the findings confirm that Gas6 knockdown can inhibit esophageal cancer progression and can exert anti-tumor effects on esophageal cancer through the PI3K/AKT pathway.

## 1. Introduction

According to the Global Cancer Statistics 2022 report [1], esophageal cancer has become the seventh leading cause of cancer death worldwide. Although the incidence and mortality rates of esophageal cancer have been decreasing in recent years, there were still 511,000 new cases and 445,000 deaths in 2022, demonstrating that esophageal cancer remains a significant burden on human health. It is of concern that esophageal cancer has become one of the top five causes of cancer death in China [2,3,4]. Among all esophageal cancer cases in China, the most common tissue subtype is esophageal squamous cell carcinoma (about 90%) [5]. Current conventional treatments for squamous esophageal cancer are still unsatisfactory, with a 5-year overall survival rate of only 15–25% [6]. Therefore, elucidating the underlying mechanisms is beneficial for the prevention and clinical targeted therapy of ESCC.

Growth-arrest-specific gene 6 (Gas6) is a secreted protein [7] involved in the development of a variety of malignant tumors [8,9]. The binding of Gas6 to TAM receptors (Tyro3, Mer, and Axl) regulates tumor cell proliferation, migration, and invasion. Gas6 can promote cancer metastasis by upregulating Slug and inhibiting E-cadherin expression [10]. Gas6 has been reported to be highly expressed in gastric cancer and associated with lymph node metastasis [11]. In addition, the overexpression of Gas6 is usually associated with poor clinical prognosis, including in ovarian, lung adenocarcinoma, pancreatic, and bladder cancers [12,13,14,15]. However, the role and mechanism of Gas6 in esophageal cancer remain largely unknown.

In this study, we aimed to reveal the role of Gas6 in esophageal cancer and elucidate the possible molecular mechanisms. Our results showed that Gas6 downregulation inhibited the proliferation, migration, and invasion of ESCC. Furthermore, bioinformatics analysis revealed that Gas6 was closely associated with PI3K family gene expression. In addition, the expression levels of P-PI3K and P-AKT decreased with the downregulation of Gas6. Thus, Gas6 knockdown may exert anti-esophageal cancer effects by inhibiting the PI3K/AKT pathway.

## 2. Materials and Methods

### 2.1. Cell Culture

The human esophageal squamous carcinoma cell lines KYSE150 and TE1 (purchased from Shanghai Cell Bank, Chinese Academy of Sciences, Shanghai, China) were cultured in RPMI1640 medium containing 10% FBS, 1% penicillin, and 1% streptomycin at 37 °C in a 5% CO_2_ incubator.

### 2.2. Lentiviral Transfection

Lentiviral vectors carrying the short hairpin RNA (shRNA) sequence 5′-CCATCCAGGAAACGGTGAAAGTGAA-3′ (Gas6) (Han Biologicals, Shanghai, China) were used to infect KYSE150 and TE1 cells. After the cells were infected for 48 h, they were maintained in 1.5 μg/mL puromycin. They were divided into the negative control group (sh-NC) and the knockdown group (sh-Gas6). The transfection effect was determined by means of qRT-PCR and Western blotting.

### 2.3. Quantitative Real-Time Polymerase Chain Reaction (qRT-PCR)

The total RNA was isolated from cultured cells using TRIzol reagent to detect Gas6 mRNA levels. The primer information is presented in Table 1.

### 2.4. Western Blotting Assay

Esophageal cancer cells in the sh-NC and sh-Gas6 groups in the logarithmic growth phase were added to the lysate (containing protease inhibitors and phosphatase inhibitors). Lysates were collected after lysis on ice, and protein concentrations were measured using the BCA assay. Then, SDS-PAGE electrophoresis was performed, the proteins were transferred to a PVDF membrane, and then the PVDF membrane was blocked. Each membrane was cut around the molecular weight of the protein of interest using the molecular weight marker as a reference, followed by incubation with primary antibodies overnight at 4 °C. The antibody dilutions were 1:1000. Gas6 (#67202), PI3K (#4292), AKT (#75692), and P-AKT (#4060) antibodies were purchased from Cell Signaling Technology (CST, Boston, MA), P-PI3K (abs130868) from Absin (Shanghai, China), and β-actin (AC026) from Abclonal (Wuhan, China). The membranes were then incubated with fluorescent secondary antibodies (Goat anti-Rabbit IgG Antibody DyLightTM 800 Conjugated-611-145-002, ROCKLAND, Limerick, PA, USA) for 2 h at room temperature and detected using the Odyssey imaging system.

### 2.5. CCK-8 Assay to Detect Cell Proliferation

Esophageal cancer cells with Gas6 knockdown were inoculated into 96-well plates at a density of 4 × 10^3^ cells/well and incubated with CCK-8 reagent for 1.5 h at 0 h, 24 h, 48 h, and 72 h. The absorbance value at 450 nm was then measured.

### 2.6. Wound Healing Experiment

Cells were inoculated into 24-well plates at 2.5 × 10^5^ cells/well. The cells were scratched once they had fully grown, and 1% FBS medium was added to continue the culture. Scratch healing was observed with Evos Auto 2 (Thermo Fisher, Bothell, WA, USA), and Image J 1.53 (National Institutes of Health, Bethesda, MD, USA) was used to measure the scratch healing area.

### 2.7. Live Cell Tracer Assay

Cells were inoculated into 96-well plates. The cells were wall-adhered and incubated with Hoechst33342 working solution for 20 min and an anti-fluorescence quencher for 2 h and then incubated with 1% FBS medium. Images were acquired in the Evos Auto 2 system every 15 min for 24 h. The results were analyzed using Celleste 6.0 imaging analysis software. Cell mobility was expressed as average movement speed and effective distance, and cell movement was monitored by time-lapse image sequences.

### 2.8. Migration and Invasion Assays

The cells were resuspended in a serum-free medium and counted so that the concentration of the cell suspension was 2.5 × 10^5^ cells/mL. In the migration assay, 100 μL of the cell suspension was added to the upper chamber of the mini-chamber, and 600 μL of medium containing 15% FBS was added to the lower chamber. The cells were fixed with 4% paraformaldehyde after 24 h of incubation, and the cells on the membrane were wiped off with a cotton swab, stained with 0.1% crystal violet solution, and photographed. In the invasion experiments, the upper chamber was coated using Matrigel (Cat. No. 356234; Corning, NY, USA).

### 2.9. Statistical Analysis

Statistical analysis was performed using GraphPad Prism 8.0 software (GraphPad Software, La Jolla, CA, USA). Differences between the two groups were determined by performing a Student’s *t*-test. A one-way analysis of variance (ANOVA) was used to analyze the differences between several groups. The results are expressed as the mean ± standard deviation. *p* < 0.05 indicates a statistically significant difference.

## 3. Results

### 3.1. Knockdown of Gas6 Expression Inhibits ESCC Cell Proliferation In Vitro

**Figure 1:**
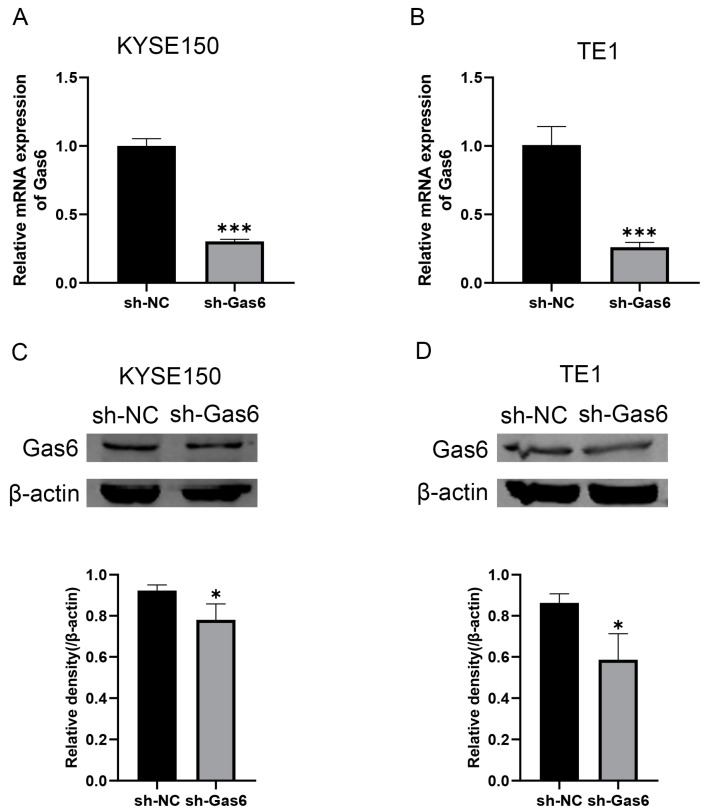
Gas6 was significantly downregulated in KYSE150 and TE1 cells. (**A**,**B**) mRNA levels of Gas6 knocked down in ESCC cells infected with Gas6 lentivirus. (**C**,**D**) Representative Western blot images and bar graphs showing Gas6 protein expression in KYSE150 and TE1 cell lines transfected with NC and sh-Gas6, *n* = 3. * *p* < 0.05, *** *p* < 0.001.

We examined the expression level of Gas6 in esophageal cancer cells by performing Western blotting, and the results showed that Gas6 was significantly expressed in KYSE150 and TE1 cells (Supplementary Figure S1), which is consistent with our previous findings [16,17]. Therefore, we downregulated Gas6 in the ESCC cell lines (KYSE150 and TE1). An evaluation using qRT-PCR and Western blotting revealed that the mRNA level and protein expression level of Gas6 were significantly decreased in the sh-Gas6 group (Figure 1), and the transfection effect was as expected.

**Figure 2:**
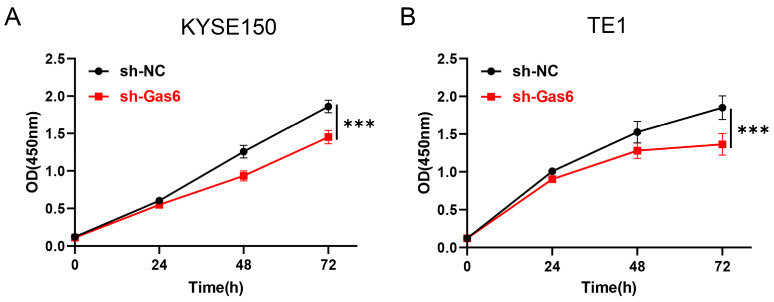
Gas6 downregulation inhibits ESCC cell proliferation. CCK-8 results showed that Gas6 knockdown inhibited the proliferative ability of KYSE150 (**A**) and TE1 (**B**) cells. Comparison with sh-NC group, *** *p* < 0.001.

The CCK-8 assay was used to detect the effect of Gas6 on cell proliferation. Compared with the control group, the transfection of sh-Gas6 significantly inhibited the proliferation of ESCC cells (Figure 2). Our results suggest that Gas6 promotes the proliferation of ESCC cells.

### 3.2. Gas6 Knockdown Inhibits Esophageal Cancer Cell Migration and Invasion

**Figure 3:**
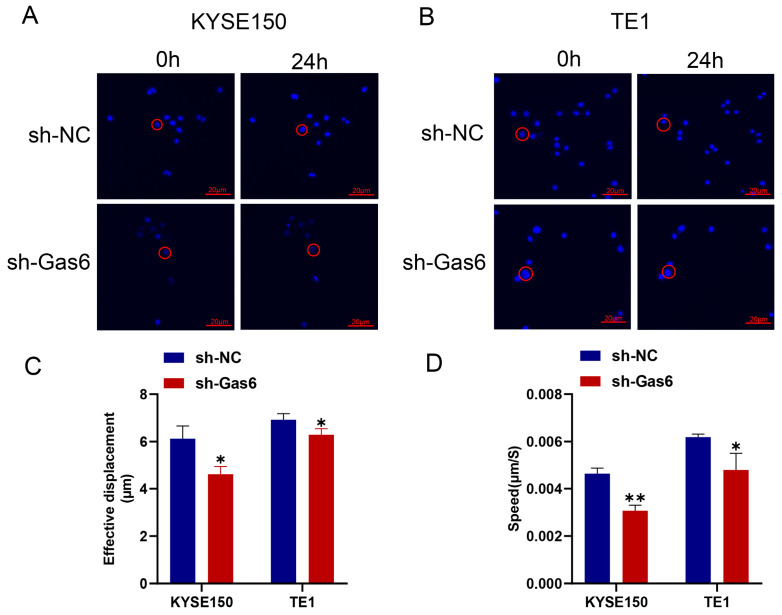
Gas6 knockdown inhibits cell moving speed and distance. (**A**,**B**) Representative fluorescent staining photographs of cell mobility. Red circles indicate the position of the same cell at 0 h and 24 h to observe the effective displacement of the cell. The figure shows that the effective displacement (**C**) and speed (**D**) of cell mobility were significantly inhibited in the sh-Gas6 group compared with the sh-NC group. * *p* < 0.05, ** *p* < 0.01.

**Figure 4:**
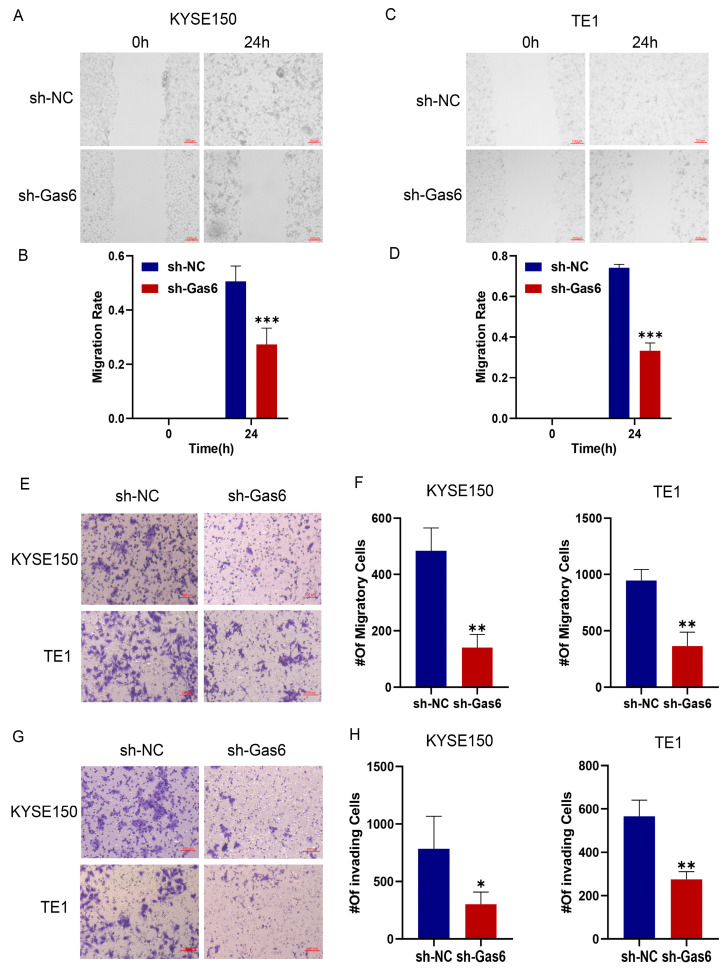
Gas6 knockdown inhibits migration and invasion of ESCC cells. (**A**–**D**) Cell scratch assay results showed that knockdown of Gas6 had an inhibitory effect on KYSE150 and TE1 cell migration. (**E**,**F**) Transwell assay showed that Gas6 knockdown significantly reduced the number of migrating esophageal cancer cells. (**G**,**H**) Invasion assay revealed that Gas6 knockdown decreased the invasion ability of KYSE150 and TE1 cells. * *p* < 0.05, ** *p* < 0.01, *** *p* < 0.001.

To examine the involvement of Gas6 in the migration of ESCC cells, we first employed a live cell tracer assay to assess cell motility, and we performed wound healing and Transwell assays to observe cell migration. The results of the live cell tracer assay showed that the position of cells in the sh-NC group was significantly changed at 24 h compared with 0 h, while the position of cells in the knockdown Gas6 group at 24 h changed less compared with 0 h (Figure 3A,B). After statistical analysis, we found that the effective moving distance of knockdown Gas6 cells was significantly shorter and the average moving speed was significantly decreased compared with the control group (Figure 3C,D). These results were obtained for both KYSE150 and TE1 cells. The results of the wound healing assay indicated that Gas6 knockdown significantly inhibited the migratory capacity of the cells compared to the control group (Figure 4A–D). Furthermore, the Transwell assay demonstrated a significantly reduced number of migrating cells in the sh-Gas6 group relative to the control group at the same time point (Figure 4E,F). In addition, we observed the effect of Gas6 on the invasion ability of ESCC by conducting an invasion assay and found that the invasion ability of KYSE150 and TE1 cells with Gas6 knockdown was significantly reduced compared with that of the control group (Figure 4G,H). The above experimental results suggest that Gas6 plays a critical role in the migration and invasion of ESCC cells.

### 3.3. Gas6 Regulates the PI3K/AKT Pathway to Promote Esophageal Cancer Progression

**Figure 5:**
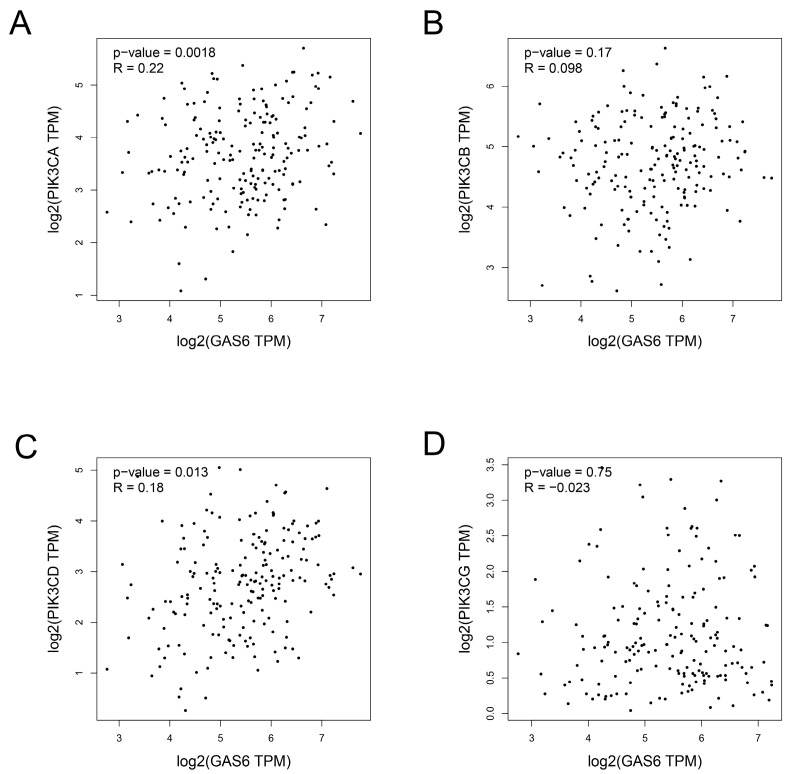
Gas6 is correlated with the PI3K gene family. (**A–D**) Scatterplot of the correlation between Gas6 expression and PIK3CA, PIK3CB, PIK3CD and PIK3CG in esophageal cancer obtained from the online tool GEPIA2. Bioinformatics showed that the expression of Gas6 gene was associated with PIK3CA and PIK3CD (all *p* < 0.05).

The PI3K/AKT pathway plays an important role in cancer metastasis and progression. To explore the relationship between Gas6 and the PI3K/AKT pathway in esophageal cancer, we examined the correlation between Gas6 and the PI3K gene family in esophageal cancer in the TCGA database with the online tool GEPIA2. The results showed that Gas6 expression was associated with PIK3CA and PIK3CD (Figure 5).

**Figure 6:**
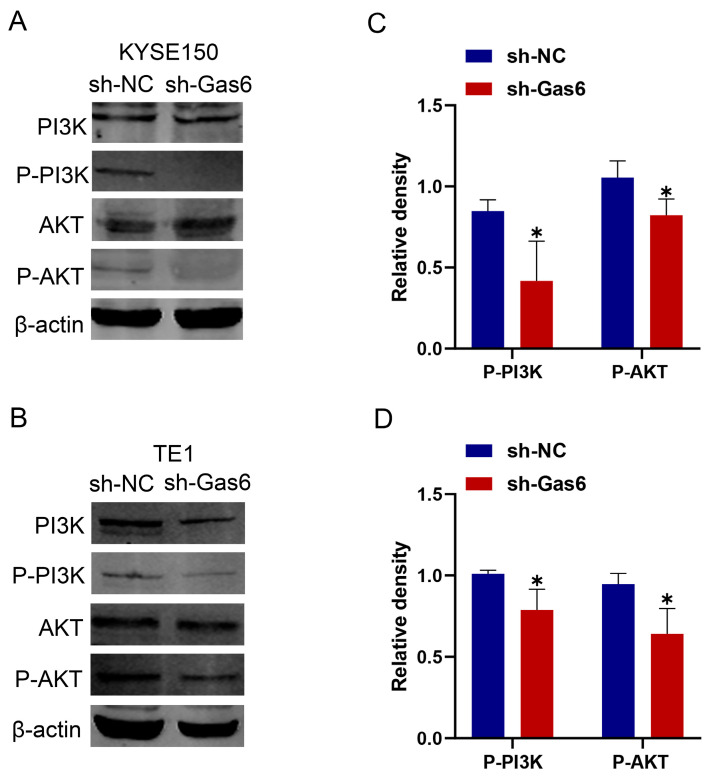
Gas6 knockdown inhibits PI3K-AKT signaling pathway. Gas6 knockdown decreased the protein expression levels of P-PI3K and P-AKT in cells. (**A**,**B**) Representative blots of PI3K, P-PI3K, AKT, and P-AKT in KYSE150 cells and TE1 cells, *n* = 3. (**C**,**D**) Statistical analysis of relative protein expression, *n* = 3. * *p* < 0.05.

In addition, we investigated the potential effects of Gas6 knockdown on PI3K/AKT-pathway-related protein expression in KYSE150 and TE1 cells. The Western blotting results showed that the expression levels of P-PI3K and P-AKT were significantly lower in the sh-Gas6 group than in the control group, and there was no statistically significant difference in the expression levels of PI3K and AKT (Figure 6). The above results suggest that Gas6 downregulation can inhibit the PI3K/AKT pathway in ESCC and exert anti-esophageal cancer effects.

## 4. Discussion

Esophageal cancer is a malignant tumor with high invasive and metastatic abilities and a poor prognosis, and elucidating its molecular mechanism is beneficial for clinical diagnosis and treatment. Here, we demonstrated that Gas6 knockdown inhibited esophageal cancer cell migration and invasion. In addition, Gas6 knockdown can inhibit the expression of PI3K/AKT-pathway-related proteins, which, in turn, exert anti-esophageal cancer effects. In summary, Gas6 can be regarded as a clinically valuable prognostic marker and a potential therapeutic target for esophageal cancer.

Gas6, a vitamin-K-dependent (VKD) protein, has been shown to be highly expressed in human tumor tissues, and high levels of Gas6 expression are associated with poorer prognosis [18]. Furthermore, Gas6 expression is upregulated in a variety of cancer cells, including ovarian cancer, glioma, and melanoma cell lines [9,19,20]. Gas6 overexpression promotes the migration and invasion of a variety of tumor cells [21,22]. The measurement of Gas6 expression may serve as a potential indicator for the diagnosis and prediction of relevant tumor progression. However, whether Gas6 has the same effect on ESCC is unclear, as is its mechanism of action.

In our team’s previous study, Gas6 was found to be highly expressed in ESCC tissues [23]. In the present study, we constructed ESCC cells with stable Gas6 knockdown via lentiviral transfection to observe the mechanism of Gas6 in esophageal cancer progression. Through a series of biological function experiments, we found that Gas6 knockdown inhibited the proliferation, migration, and invasion of ESCC cells.

Although we have experimentally identified the effects of knocking down Gas6 on ESCC migration and invasion functions, the molecular mechanisms underlying the role of Gas6 in ESCC remain unclear. Previous studies have shown that Gas6 is able to regulate tumor cell migration and invasion through the JAK2/ERK, SRC, and Akt/GSK-3β/β-catenin pathways [24,25,26]. In addition, it has been shown that Gas6 is highly expressed in bladder cancer and is significantly correlated with PI3K expression. Several studies have shown that the PI3K/AKT signaling pathway is involved in esophageal cancer progression [27,28]. The PI3K/AKT pathway, as a classical pathway regulating the proliferation, metastasis, and invasion of tumor cells, has attracted our attention [15,29,30,31,32]. Therefore, we speculated that Gas6 might play a pro-esophageal cancer role through the PI3K/AKT pathway. In the present study, we first examined the correlation between Gas6 and PI3K family genes in the TCGA database by using the online tool GEPIA2 and found that Gas6 was closely related to PI3K family genes, which is consistent with a previous study [15]. Then, we further verified the effect of Gas6 knockdown on the levels of PI3K, AKT, and the phosphorylated forms of both proteins. The results showed that there was no significant change in the total protein expression of PI3K and AKT, and the protein levels of P-PI3K and P-AKT clearly decreased with Gas6 knockdown. These results suggest that Gas6 knockdown can exert anti-esophageal cancer effects through the PI3K/AKT pathway.

However, although this study analyzed the role of Gas6 in ESCC, confirmed that Gas6 knockdown may inhibit esophageal cancer migration and invasion through the PI3K/AKT pathway, and provided relevant evidence, its limitations still need to be acknowledged. Due to certain differences between the in vitro and in vivo environments, the relevant results offer limited clinical guidance. Moreover, it is necessary to design reasonable protocols to carry out in vivo studies in the future to explore the role of Gas6 and related molecular mechanisms in esophageal cancer in a more complex environment so as to more powerfully validate and expand the current findings and to provide the possibility of future application in clinical diagnosis and treatment.

## 5. Conclusions

**Figure 7:**
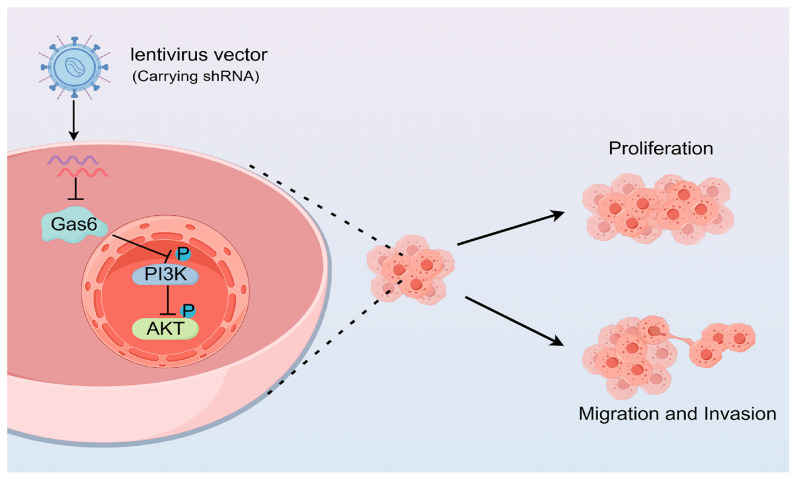
Schematic representation of Gas6 knockdown inhibiting PI3K-AKT pathway and affecting ESCC cell proliferation, migration and invasion.

Our study demonstrated that Gas6 is able to modulate the PI3K/AKT pathway to affect the proliferation, migration, and invasion of ESCC cells and plays a key role in this process (Figure 7).

## Figures and Tables

**Table 1:** Primers used for qRT-PCR analysis of gene expression.

Gene	Primer Sequence 5′ > 3′	
	Forward	Reverse
**Gas6**	CATCCAGGAAACGGTGAAA	GGAGTGATAGTCTACCAGTGCC
**GAPDH**	TCAAGGCTGAGAACGGGAAG	TCGCCCCACTTGATTTTGGA

## Data Availability

The original contributions presented in the study are included in the article; further inquiries can be directed to the corresponding author.

## Supplementary Materials

The following supporting information can be downloaded at: https://www.mdpi.com/article/10.3390/cimb46100676/s1, Figure S1. Expression of Gas6 protein in immortalized esophageal epithelial cells SHEE and esophageal cancer cells KYSE150, ECA109 and TE1.The expression of Gas6 was obvious in KYSE150 and TE1. Figure S2. The original WB Blots of Figure 1C,D. Protein expression of Gas6 in the KYSE150 and TE1 cell lines transfected with NC and sh-Gas6 (n=3). Figure S3. The original WB Blots of Figure 6A. Protein expression of PI3K, P-PI3K, AKT, and P-AKT in KYSE150 cells transfected with NC and sh-Gas6 (n=3). Figure S4. The original WB Blots of Figure 6B. Protein expression of PI3K, P-PI3K, AKT, and P-AKT in KYSE150 cells transfected with NC and sh-Gas6 (n=3).The PI3K and AKT were incubated after elution of P-PI3K and P-AKT by the eluent, so that the β-actin of total and phosphorylated proteins were consistent. Figure S5. The original WB Blots of Figure S1. Expression of Gas6 protein in immortalized esophageal epithelial cells SHEE and esophageal cancer cells KYSE150, ECA109 and TE1.
