# Supplementary material for: Knockdown of Gas6 Exerts Anti-Esophageal Cancer Effects by Inhibiting the PI3K/AKT Pathway
Source: Curr Issues Mol Biol. 2024 Oct 13;46(10):11349–58. doi: 10.3390/cimb46100676 (PMC11506498; doi:10.3390/cimb46100676)
Supplement: Supplementary file 1 [file cimb-46-00676-s001.zip › cimb-3232823-Supplementary Materials.pdf]

Supplementary Materials

Knockdown of Gas6 exerts anti-esophageal cancer effects by inhibiting the PI3K/AKT pathway

Shuang Gao [1, 2], Yu Wang [1, 2], Ming Huang [1, 2], Jianxin Guo [1], Zhongbing Wu [1,*] and Jing Li [1,*]

1 College of Integrated Chinese and Western Medicine, Hebei Medical University, Shijiazhuang 050017, China

2 Institute of Integrated Traditional Chinese and Western Medicine, Hebei Medical University, Shijia-zhuang 050017, China

* Correspondence: J.L., E-mail: lijing@hebmu.edu.cn; Z.B.W., E-mail:19001631@hebmu.edu.cn

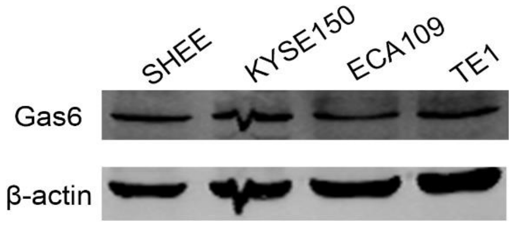

Figure S1. Expression of Gas6 protein in immortalized esophageal epithelial cells SHEE and esophageal cancer cells KYSE150, ECA109 and TE1.The expression of Gas6 was obvious in KYSE150 and TE1.

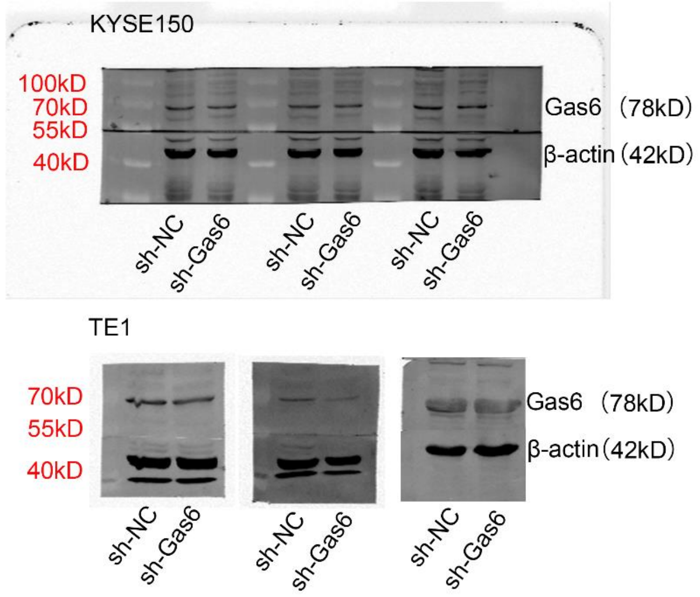

Figure S2. The original WB Blots of Figure 1C and 1D. Protein expression of Gas6 in the KYSE150 and TE1 cell lines transfected with NC and sh-Gas6(n=3).

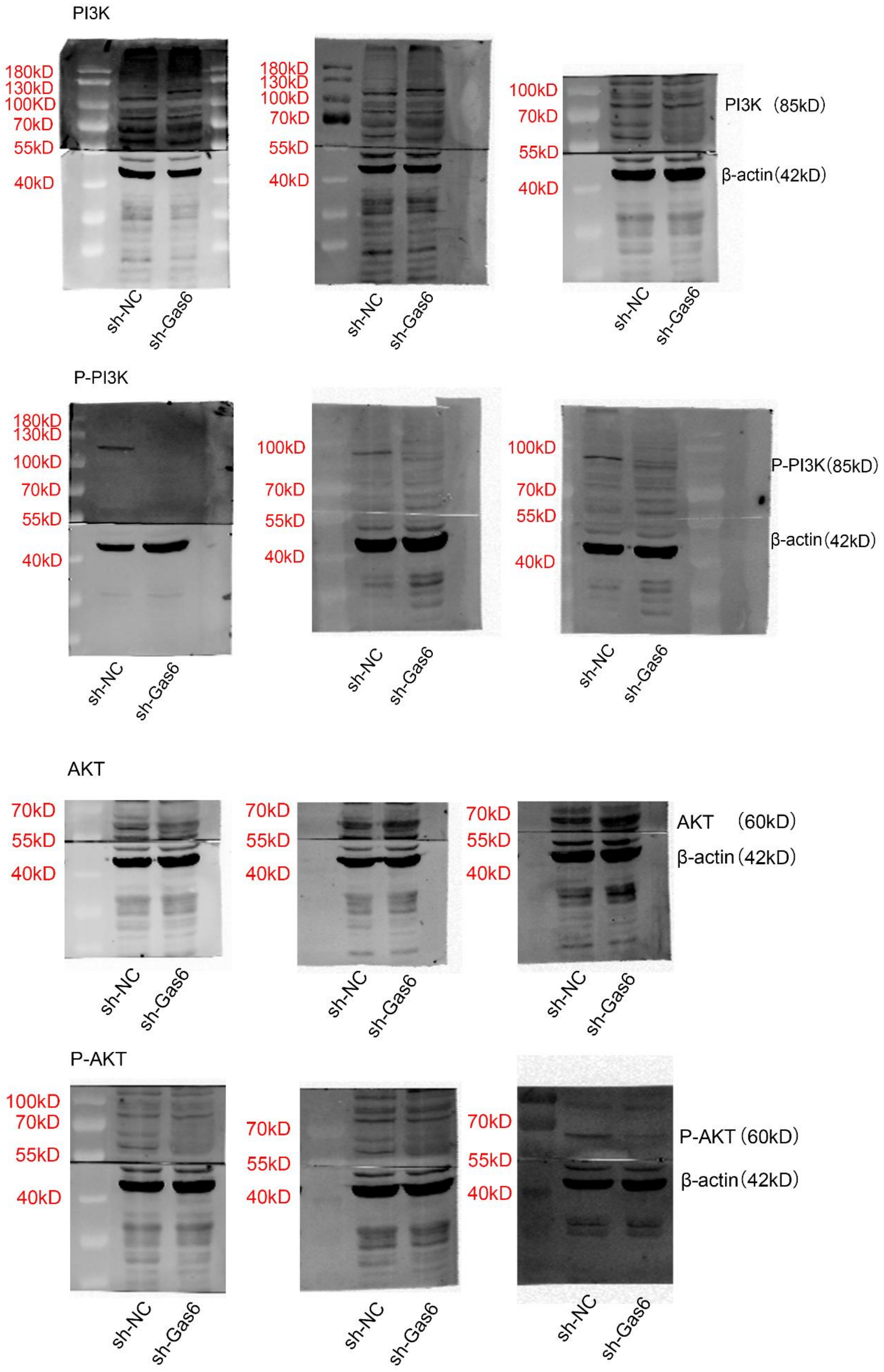

Figure S3. The original WB Blots of Figure 6A. Protein expression of PI3K, P-PI3K, AKT, and P-AKT in KYSE150 cells transfected with NC and sh-Gas6(n=3).

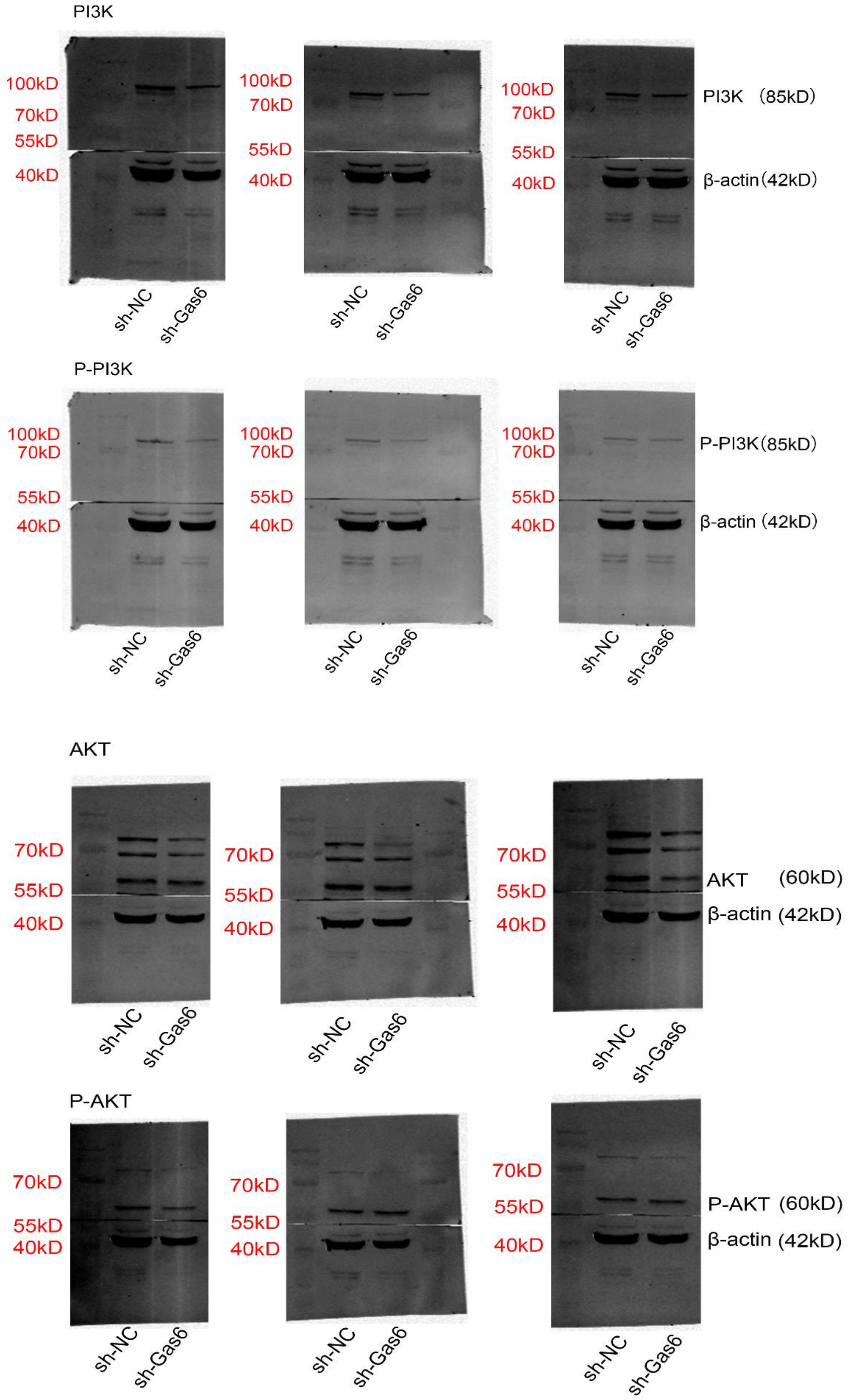

Figure S4. The original WB Blots of Figure 6B. Protein expression of PI3K, P-PI3K, AKT, and P-AKT in KYSE150 cells transfected with NC and sh-Gas6(n=3).The PI3K and AKT were

incubated after elution of P-PI3K and P-AKT by the eluent, so that the β-actin of total and phosphorylated proteins were consistent.

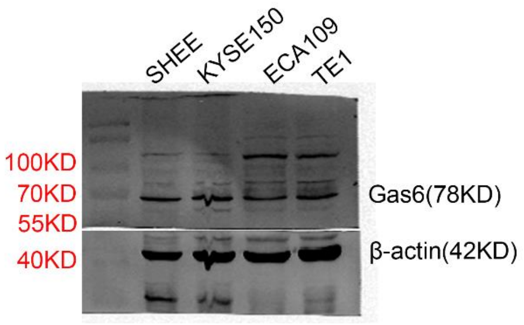

Figure S5. The original WB Blots of Figure S1. Expression of Gas6 protein in immortalized esophageal epithelial cells SHEE and esophageal cancer cells KYSE150, ECA109 and TE1.
